# Game-Based Approaches’ Pedagogical Principles: Exploring Task Constraints in Youth Soccer

**DOI:** 10.1515/hukin-2015-0053

**Published:** 2015-07-10

**Authors:** Jaime Serra-Olivares, Sixto González-Víllora, Luis Miguel García-López, Duarte Araújo

**Affiliations:** 1 Departamento de Pedagogía en Educación Física. Facultad de Educación. Universidad Católica de Temuco. Temuco, Chile.; 2 Facultad de Educación. Universidad de Castilla-La Mancha. Cuenca, España.; 3 Facultad de Educación. Universidad de Castilla-La Mancha. Albacete, España.; 4 CIPER, Faculdade de Motricidade Humana, SpertLab. Universidade de Lisboa. Lisboa Portugal.

**Keywords:** game-based approaches, degeneracy processes, game performance, small-sided games

## Abstract

This study tested the use of two pedagogical principles of Game-based approaches, representation and exaggeration, in the context of game performance of U10 soccer players. Twenty-one players participated in two 3 vs. 3 small-sided games. The first small-sided game was modified by representation. The second small-sided game was modified by enhancing the penetration of the defense tactical problem for invasion games. Decision-making and execution were assessed using the Game Performance Evaluation Tool. No significant differences were observed between games in the number of decision-making units related to keeping possession, nor in those related to penetrating the defense. No significant differences were observed in any execution ability (ball control, passing, dribbling and get free movements). The findings suggested that both games could provide similar degeneracy processes to the players for skill acquisition (specific and contextualized task constraints in which they could develop their game performance and the capability to achieve different outcomes in varying contexts). Probably both games had similar learner-environment dynamics leading players to develop their capabilities for adapting their behaviours to the changing performance situations. More research is necessary, from the ecological dynamics point of view, to determine how we should use small-sided games in Game-based approaches.

## Introduction

Pedagogical principles of Game-based approaches (GBAs) are one of the foundations of the games curriculum model for developing decision-making and skill performance in games (Bunker and Thorpe, 1982; Thorpe et al., 1986). The main four pedagogical principles of GBAs are sampling, tactical complexity, representation and exaggeration. They were based on the student-based approach that teaches tactical awareness and skills through small-sided games (SSGs), leading learners to improve their knowledge and skills in a more innovative learning context. There have been many attempts to provide appropriate teaching materials for curriculum development (e.g., modified games to facilitate student learning (Mitchell et al., 2006)). However, it is not clear how pedagogical principles influence tactical behaviours differently, as task constraints. From an ecological point of view, tactical behaviours consist of intentional adaptations to the environmental constraints imposed by the specific context of play during task performance (e.g., Araújo et al., 2006; Araújo and Davids, 2009; Travassos et al., 2012). In that regard, for a specific task the performer and his or her environment are a pair of dynamical sub-systems that are coupled and that interact mechanically and informationally. For this reason, it is important to understand the coordination of a performer and his/her environment within the process of teaching and learning motor skills (Passos et al., 2008; Renshaw et al., 2007; Renshaw et al., 2009). Seifert et al. (2013) suggested that individuals, as complex and dynamical systems in degeneracy processes (Whitacre and Bender, 2010), adapt their motor actions and coordinate their degrees of freedom using among others such factors as: ‘multi-stability’ (i.e. the ability to transit between multiple states of organisation under given constraints), ‘meta-stability’ (i.e. the ability to exploit coexisting coordination tendencies in a transition or unstable region) and ‘variability’ properties (the exploitation of critical fluctuations to enable adaptive behavioural transitions). “Degeneracy signifies that an individual can vary motor behaviour (structurally) without compromising function, providing evidence for the adaptive and functional role of movement pattern variability in order to satisfy task constraints. The presence of degeneracy in a biological system increases its complexity and robustness against perturbation and underlies ‘pluripotentiality’, a property that ensures an organism’s functional ongoing engagement with the dynamic performance environment” (Seifert et al., 2013, p. 173). Coordinating degrees of freedom in assembling actions is essential to ensure the ability of elements that are structurally different to perform the same function. It suggests that team games tactical behaviours should be trained taking into account the inherent adaptive flexibility in achieving successful performance outcomes of neurobiological systems (athletes), including social neurobiological systems (sports teams) (Vilar et al., 2012). Taking into account the performer/team/context relationship during the task design would lead learners to achieve a higher level of performance (decision-making and skill execution), by emphasizing the potential adaptation of human movements. In this sense, representative training tasks should be designed within the teaching games process (Pinder et al., 2011).

Nonlinear Pedagogy, as a part of the ecological dynamic approach, has provided a relevant framework for modeling athletic performance and youth sports. Nonlinear Pedagogy explains how GBAs such as Teaching Games for Understanding (TGfU) (Bunker and Thorpe, 1982; Thorpe et al., 1986), might support learning concepts for games teaching and coaching. It is useful in the understanding of how movement changes, and provides information regarding youth’s readiness to acquire and develop game skills. Indeed, it shows how teachers and coaches could use task, environmental and performer constraints to guide the process of skills acquisition and decision-making in learners (Renshaw et al., 2009; Renshaw et al., 2010). Thus, it is important to derive, theoretically, the representativeness of the sport tasks being studied (Aguiar et al., 2012; Almeida et al., 2013; Chow and Tan, 2009; Passos et al., 2008; Pinder et al., 2011; Richard et al., 2005; Travassos et al., 2012). The representative tasks design in GBAs is based on the four principles mentioned above in the introduction (sampling, tactical complexity, representation and exaggeration) (Tan et al., 2012). Representation implies the use of SSGs as ecological tasks that have the same structure as the official game, but the size of the elements of play is reduced. In this environments based on the mutuality of the performer and the environment, learners can attune their movements to the essential information through practice, and this processes help them to establish strong ‘information–movement couplings’ to guide their behaviours (Renshaw et al., 2007, 2009, 2010). For example, in mini-basketball, there is no three-point line and the areas of play are reduced, so its tactical complexity is assumed to be similar to that of the official game of basketball and adapted to the learners’ characteristics. Alternatively, the pedagogical principle of exaggeration involves the modification of key elements of play to provide learners with the opportunity to explore specific tactical problems while maintaining the primary rules of the game. For example, if the goals in soccer are removed, the tactical problem of how to keep the ball, using passes and get-free movements, will be enhanced. However, while pedagogical literature explains how to modify games (e. g., Harvey and Jarrett, 2013; Mitchell et al. 2006), few scientific studies have provided justifications that support the modification of task constraints using the pedagogical principles of GBAs within the representative design of games (e. g., Almeida et al., 2012, 2013; Da Silva et al., 2011; Lapresa et al., 2010; Teoldo et al., 2010; Travassos et al., 2012). Therefore, what are the consequences of every modified game? Why should coaches use one SSG or another in teaching games? How should coaches use the GBAs’ pedagogical principles of representation and exaggeration in teaching invasion games? How can coaches induce functional interactions between a player and a context?

In relation to the above, we found some studies in which the flexibility and adaptability of game performance (decision-making and movement skills) were assessed, taking into account the number of players that participated in the task, the pitch dimensions of the game, and more importantly, the influence of the tactical context constraints of the game (Griffin et al., 1995; Mitchell et al, 1995). Some studies such as those performed by González-Víllora et al. (2011) or Gutiérrez et al. (2011) observed that players’ behaviours were more influenced by tactical problems (in attack: keeping possession, penetrating the defense and attacking the goal), as defined by Bayer (1992), than by the number of players or by field sizes. Serra-Olivares et al. (2011) compared two 3 vs. 3 SSGs that were modified by pedagogical principles. One SSG was modified by representation and another was modified by the pedagogical principles of representation and exaggeration. In that study, the tactical problem of keeping possession of the ball was enhanced. In the game that exaggerated this tactical aspect, the authors observed a significantly greater number of situations of keeping the ball and a better adaptation of the players to the tactical contexts. However, the players made more successful decisions and executions in the SSG in which the pedagogical principle of representation was used.

Correia et al. (2012) argued that it is essential to assess athletic performance in stable and unstable relationships of the performer and the environment (such as SSGs modified by the GBAs’ pedagogical principles of representation and exaggeration). These experiences facilitate the convergence to more functional (effective) couplings of information and movement by the sport learner (Renshaw et al., 2009). Consequently, it seems essential to research the pedagogical principles of GBAs in order to understand the development of individual-environment relations through a representative tasks design in sports (Chow and Tan, 2009; Pinder et al., 2011; Renshaw et al., 2007; Tan et al., 2013). Therefore, the purpose of this research was to analyse how the game performance of young soccer players is influenced by the GBAs’ pedagogical principles of representation and exaggeration. Specifically, the aim was to examine the influence of exaggerating the tactical problem of penetrating the defense on tactical context-adaptation and the game performance of the players, as degeneracy processes within biological systems.

## Material and Methods

### Participants

Twenty-one U10-skilled soccer players (age: 8.7 ± 0.3) participated in this research. They belonged to four Under-10 teams of the Spanish Soccer Club Academy. All of them had engaged in at least one year with more than three hours per week of specific practice in soccer and had experience in regional competitions. They were selected by their coaches as the best performers on their teams. Players’ parents signed an informed consent form allowing their children to participate. This work was approved by the University of Castilla-La Mancha ethics committee before data collection commenced.

### Measures

The Game Performance Evaluation Tool (GPET) is an observational system for notational analysis (García-López et al., 2013), it offers the possibility of analyzing each decision made from the tactical viewpoint of the problem the player has to solve in the game play he is involved in. It differs from the GPAI (Oslin et al., 1998) and the TSAP (Gréhaigne et al., 1997) as they assess decision making and skill execution, but the result of their analyses is not related to the tactical problems in which decisions and executions take place (Memmert and Harvey, 2008). In the GPET, game performance is categorised into three dimensions of the variable: tactical context-adaptation performance, decision-making and skill execution (Table 1).

Related to tactical context-adaptation in GPET, players’ tactical intentions are coded with regard to the principal tactical problem in attack in which the action is located (Bayer, 1992; Gréhaigne and Godbout, 1995; Mitchell et al., 2006): keeping possession of the ball, penetrating the defense and attacking the goal. They are coded as 1 (Correct) and 0 (Incorrect). The “watcher-player” behaviour is also analysed in this dimension. In the second dimension, decision-making skills are grouped by the game roles of attacking, which could be as on-ball players and as off-ball players, but they are also coded as 1 (Correct) and 0 (Incorrect) with regard to the tactical problems for the invasion game in which a player is located. The skill execution component is coded as 1 (Success) or 0 (No success). For assessment purposes, in GPET playing time is divided into decision-making units (Nevett et al., 2001), as in previous research (González-Víllora et al., 2012; Gutiérrez et al., 2011). A decision-making unit ends after 4 s of action, when the player performs a different technical-tactical skill, or when the tactical problem changes. This instrument has been validated by García-López et al. (2013), with appropriate intra- and inter-observer correlations in all categories. It has been shown to be a reliable tool for game performance assessment (*α*=.97). Indeed, the observer of the present study was trained in the instrument and showed intra-observer correlations that were similar to the correlations of García-López et al. (2013), ranging from .77 to 1.00, in all categories of the instrument.

### Procedures

**Figure 1:**
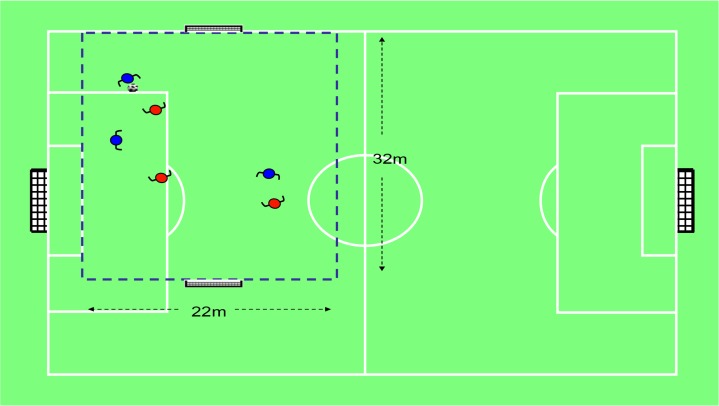
The 3 vs. 3 SSG modified by the pedagogical principle of representation (SSG-R) The rules are similar to the adult parent game of soccer, although there are no goalkeepers. It is played in an area of 32 × 22 meters. The main objective is to score as many points as possible. One point is scored when one player kicks the ball into the opposing team’s goal. Each team defends its own goal and attacks the opposing team’s goal (140 × 105 centimetres). Attackers are allowed to control, pass, dribble, kick and support (get-free) during the game. Kicking from a player’s own field is not allowed.

**Figure 2:**
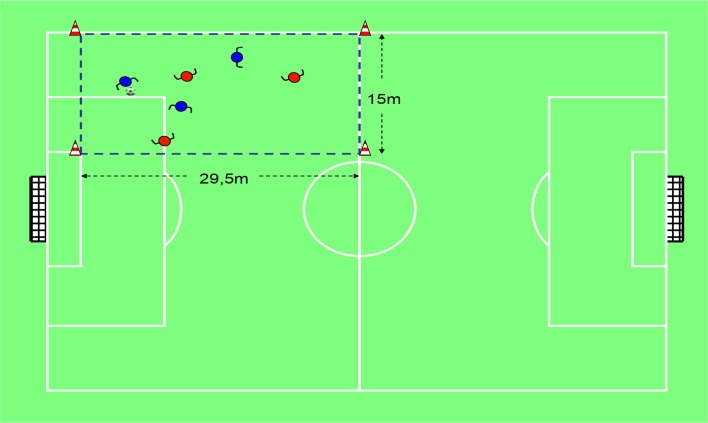
The 3 vs 3 SSG modified by the pedagogical principles of representation and exaggeration (SSG-R&E) This game is focused on the tactical problem of penetrating the defense. It is played in an area of 29.5 × 15 meters. The main objective is to score as many points as possible. One point is scored when an offensive player receives the ball from a teammate behind the opposing team’s goal (an imaginary line of 15 meters between two cones). Each team defends its own goal and attacks the opposing team’s goal. Attackers are allowed to control, pass, dribble, kick and support (get-free) during the game. Dribbling to advance to the opposing goal is forbidden.

A comparative study was designed. The participants were assessed in two different SSGs. The order in which they played these games was randomised. The first game was modified by representation (Figure 1), and the second game was modified by the pedagogical principles of representation and exaggeration (Thorpe et al., 1986). The second game highlighted the tactical problem of “penetrating the defense” (Figure 2). The two games lasted for 8 min, divided into two halves. In this sense, we analysed the influence of the tactical problems in invasion games (Bayer, 1992) as task constraints on the tactical behaviour of the players, that is, as an example of the degeneracy processes of dynamical systems.

Participants were organised in seven teams of three players. Seven 3 vs. 3 matches (8 min each) were video recorded for each of the two SSGs designed for this research (the SSG-R and the SSG-R&E). Prior to the matches, the players participated in a similar warm-up consisting of general mobility and stretching exercises. Then, the game rules were explained to them, and they practiced the game during the minutes before the recording. The players’ game performance was codified in the SSG-R and the SSG-R&E using the GPET.

### Statistical Analysis

The means and standard deviations were calculated for all dimensions of the variable in each of the SSGs that was recorded. Then, the game performance of the players, i.e. tactical context-adaptation performance, technical-tactical skill decision-making and execution dimensions, were compared between games. The *Kolgomorov-Smirnov* test for the assumption of normality and the *Levene* test for the homogeneity of variance showed that the sample did not meet these assumptions for all of the variables. Therefore, the *Wilcoxon* test was conducted to analyse the differences between the players’ game performance in the two SSGs.

## Results

The game performance of the 21 soccer players in the SSG-R and SSG-R&E modified games was compared in line with two of the three tactical problems in attack for invasion games that were proposed by Bayer (1992): keeping possession of the ball and penetrating the defense. We did not compare the game performance of the third tactical problem (attacking the goal) with the previous two, as there were no kicks in the SSG-R&E: in that modified game, there were no goals. We analysed 1695 decision-making units: 887 in the SSG-R (17.7% in keeping possession of the ball, 76.7% in penetrating the defense and 5.5% in attacking the goal situations) and 808 decision-making units in the SSG-R&E (16.4% in keeping possession of the ball situations and 83.6% in penetrating the defense situations). The results are summarised by game modification and compare the tactical context-adaptation performance, decision-making and skill execution components of game performance within each tactical problem (Table 2).

No significant differences between the two SSGs were found in the number of decision-making units related to the tactical problems of keeping possession of the ball (*Z=* −.243*. p=* .80) or penetrating the defense (*Z*= −.296. *p*= .76). Related to tactical context-adaptation, the soccer players scored slightly better in both tactical problems in the SSG-R&E, although the differences were not significant. There were also no significant differences in the decision-making and skill execution dimensions of game performance in any skill (ball control, passing, dribbling, and get-free movements). The differences were only significant in the watcher-player variable. In this sense, the soccer players scored significantly higher in the SSG-R&E. The analysis showed that players’ game performance was similar between games, although the decisions and executions of the get-free movements to keep the ball were lower in the SSG-R&E (*Z*= −1.76. *p*= 0.07) and (*Z*= −1.76. *p*= 0.07), respectively.

## Discussion

The purpose of this research was to analyse how the exaggeration of penetrating the defense tactical problem influenced youth soccer players’ game performance in two 3 vs. 3 SSGs. Findings showed that both games could be used to teach some specific attacking concepts related to invasion games tactical problems. This research differs from others in which only games modified using the principle of representation were studied, and in which the SSGs’ complex dynamics and the degeneracy processes of biological systems were not analysed in accordance with the ecological perspective (e.g. Aguiar et al., 2012; Lapresa et al., 2010; Teoldo et al., 2010). In this sense, the results suggested that both of the games analysed could provide similar processes for the acquisition of skills to these players, although the SSG-R may be slightly more tactically complex. First, no significant differences between the games were found in the number of decision-making units observed in each tactical problem, either in tactical context adaptation related to keeping possession of the ball or in tactical problems of penetrating the defense. These results differ from those observed by Serra-Olivares et al. (2011), who compared a similar 3 vs. 3 SSG that was modified by representation with a SSG in which the tactical problem of keeping the ball in possession was exaggerated. In that study, a significantly higher number of situations of keeping the ball were observed in the modified game that enhanced this problem.

On the other hand, the distribution of the decision-making units in each tactical problem in the SSGs that were analysed in this study was similar to that in other research (González-Víllora et al., 2010, 2011, 2012; Gutiérrez et al., 2011). In those studies, the game performance of youth soccer players was assessed in SSGs (from 2 vs. 2 to 5 vs. 5) that were modified using the pedagogical principle of representation, and the proportion of penetrating the defense decision-making units was higher than the proportion of units for problems of keeping the ball and attacking the goal. These findings suggest that the combined use of the pedagogical principles of representation and exaggeration used in this study to guide the game objective to penetrating the defense could provide learners with similar relationships between players and context for degeneracy processes as situations in the parent game of soccer. In this sense, it is probable that the use of SSGs in which penetrating the defense is enhanced could facilitate practice situations with greater transfer of expertise than SSGs in which the players only had to keep the ball. These aspects connect with the assumption that the transfer of expertise in sports can be defined as the amount of cooperation or competition between the dynamics of each individual and the task dynamics. Seifert et al. (2013) suggested that, when the gap between the dynamics of a learner and the task demands is low (as in the SSGs analysed here), and/or when the demands of the task are weak, convergence between both elements might be expected to facilitate the transfer of skills. This is an aspect that must be taken into account by those who are in charge of teaching sports (Gréhaigne and Godbout, 1995).

In this regard, it is important to know how principles of ecological psychology and dynamical systems theory can underpin a philosophy of coaching practice within a nonlinear pedagogy in the design of SSGs (Renshaw et al., 2009). In line with the framework presented by Araújo et al. (2006), this paper clarified how game performance could be understood as an integral part of the game tactical behaviours that are influenced by technical-tactical constraints at the scale of the environment-player relationships (e.g. Almeida et al., 2013; Memmert and Harvey, 2008; Passos et al., 2008; Vilar et al., 2012). In the future, more studies should be performed to understand how to use the pedagogical principles of GBAs to design tasks that provide similar ecological constraints to game dynamics (Gréhaigne and Godbout, 1995; Pinder et al., 2011), in order to facilitate the development of the multi-stability and meta-stability behavioural properties of the learners (Araújo and Davids, 2009; Renshaw et al., 2007; Tan et al., 2012; Kelso, 2012). In that regard, arguing that both game demands were related differently to tactical context adaptation was an exception. The players scored significantly higher in the watcher-player variable in the SSG-R&E. They showed very similar values to the students observed by Gutiérrez et al. (2011). However, it is likely that our results were not primarily due to the self-centred personalities and limited attention spans of the players, as suggested by those authors. It can be stated that the players in this study were more influenced by the specific game modifications (GBAs’ pedagogical principles) that provided specific dynamical contexts and task constraints (e.g., differences between games in get-free movements to keep the possession of the ball were close to be significant; in the game that enhances penetrating the defense tactical problem players wanted to achieve points as soon as possible going forward, forgetting to support teammates to improve team game). It provides evidence of the adaptive and functional role of movement pattern variability to satisfy task constraints in a degeneracy process (Renshaw et al., 2007, 2009, 2010; Seifert et al., 2013).

On one hand, these findings highlight that players had more difficulty in deciding what to do in some situations in the SSG-R&E, which provides evidence of behavioural flexibility and is probably due in this study to the specific task constraints of the game, as it has been observed also in other studies. For example, the width of the field was lower in the SSG-R&E analysed here, which facilitated the emergence of tactical behaviours by the defenders to keeping the attack away from the target. On the other hand, the results indicate that the same tactical problems, as constraints of these dynamical sub-systems, could have affected the game performance skills in different ways. For example, players had to develop dribbling and get-free decision-making skills to advance to the opposite goal in several situations of the games. However, the informational constraints were different depending on the game played and its ecology. This factor supports the assumption of the behavioural variability and adaptability of the players when ecological constraints are altered and action rules of the game are affected (Gréhaigne and Godbout, 1995; Pinder et al., 2011; Renshaw et al., 2007). This aspect was observed for example in the get-free game performance analysis. The players had much more difficulty in keeping the ball and in penetrating the defense using the get-free skill in the SSG-R&E, which indicates that the specific task constraints of this game affected the off-ball movements in a different way. Although these differences were not significant, it is important to highlight this trend.

One of the limitations of this study is that it did not register the defense phase of play. Defensing actions would presumably affect the results, as the attack and the defense coadaptative behaviors continually interact affecting the players-team purposes in an invasion game such as soccer or basketball (Bayer, 1992). The main defense purpose in each SSG that was designed here was different, in the SSG-R the defense of the own-goal is fundamental while in the SSG-R&E the defense should focus on not allowing the attackers to penetrate the defense (not to advance space) (Mitchell et al., 2006). This change in the strategies to be performed is easy for an expert to appreciate, but the question is whether or not it is equally simple for inexperienced young players. Its aspects have been taken into account in other studies that assessed the game performance of youth soccer players in SSGs using the pedagogical principle of representation in Under-8 players in 2 vs. 2 (González-Víllora et al., 2012), in Under-10 in 3 vs. 3 (González-Víllora et al., 2011), and in Under-12 in 5 vs. 5 SSGs (González-Víllora et al., 2010). Therefore, physical education teachers and coaches should consider that tactical problems could be a key tool to design quality ecological tasks that facilitate degeneracy processes when they are using the pedagogical principles of the GBAs. However, whereas different authors (e. g. Harvey and Jarrett, 2013; Mitchell et al., 2006; Thorpe et al., 1986) have suggested that it is advisable to develop a contextualised treatment of the basic tactical problems in the sport initiation stages (novice practice), it is unclear how we should use the pedagogical principles of the GBAs. For example, questions such as: which tactical problems should be given priority over others? And, which tactical problems should be taught first?, remain to be explored.

Due to all the considerations mentioned above, this study differs from others in which the technical-tactical difficulties of youth players were only assessed as task constraints according to the specific rules of the games (Almeida et al., 2012; Arias et al., 2011), the numbers of players who played (Da Silva et al., 2011), the different goal sizes (Teoldo et al., 2010) an the different field sizes (Gutiérrez et al., 2011; Lapresa et al., 2010). This study showed that the variability and flexibility of the behaviours during invasion games are more affected by the specific tactical constraints of the game and the internal degeneracy processes of the players. They are more influenced by the kinds of relationships between players and a context that they must face, as Renshaw et al. (2009) suggest, which is maybe produced by the specific use of the pedagogical principles of representation and exaggeration. In this sense, taking into account some of the premises of the ecological model of decision-making: a) decision-making is strongly influenced by the detection and use of contextual information, b) the acquisition of decision-making skills is characterised by the progressive perceptual attunement to relevant sources of information, and c) it is possible to capture stable patterns of interaction between performers and their environment (Araújo et al., 2006). It seems necessary to perform research regarding the ecology of the SSGs, as well as the variability and adaptability of sports behaviours, in order to effectively use the GBAs’ pedagogical principles of representation and exaggeration. In conclusion, our findings are similar to those of Pinder et al. (2011), and show that it is necessary to analyse experimental tasks (e.g. modified games or competition forms), considering the representative design of the performance context. It may allow a correct diagnosis of the critical aspects of performance required to be trained or enhanced, and the development of intervention or training tasks that achieve those goals.

## Figures and Tables

**Table 1:** Game performance dimensions in the GPET

Tactical context-adaptation performance – Tactical context-adaptation for keeping the ball problems: Efficiency in selecting actions to keep the ball when the tactical problem is coded as “keeping-the-ball context”. – Tactical context-adaptation performance for penetrating the defense problems: Efficiency in selecting actions to advance to the opposing goal when the tactical problem is coded as “penetrating the defense context”. – Tactical context-adaptation performance for attacking the goal problems: Efficiency in selecting actions to try to score when the tactical context is coded as “attacking the goal context”. – Watcher-player: A player is coded as a “watcher-player” when he does not show tactical intention or involvement in the game.	
Decision-making:	
Attacker on the ball: Pass decision-making Dribbling decision-making Kick decision-making	Attacker off the ball: Get-free skills decision-making
Skill execution	
Attacker on the ball: Pass execution Dribbling execution Kick execution	Attacker off the ball: Get-free skills execution

Decision-making and skill execution variables in attacking the goal contexts were not codified in the SSG-R&E that was analysed in this study, because there were no opportunities to score in this game

**Table 2:** Differences in the 21 players’ game performance between the SSG-R and the SSG-R&E

	SSG-R		SSG-R&E			

Dimensions	*M*	*SD*	*M*	*SD*	*Z*	*p*
	
Tactical problems of context-adaptation to keep the ball	84.00	18.63	86.66	19.92	−0.69	.49
Tactical problems of context-adaptation performance to penetrating the defense	82.91	11.56	84.22	11.84	−1.09	.27
Watcher-player	1.70	2.07	5.97	6.35	−3.77	.00
Game performance in keeping possession of the ball:						
Ball control	87.27	14.59	85.22	16.37	−0.34	.73
Pass decision-making	90.47	26.23	97.12	22.23	−0.96	.33
Dribbling decision-making	100	-	100	-	−0.44	.65
Get-free movements decision-making	93.75	17.67	45.63	36.79	−1.76	.07
Pass execution	76.87	39.35	79.22	31.15	−0.70	.48
Dribbling execution	80.35	34.02	83.97	30.13	−0.44	.65
Get-free movements execution	93.75	17.67	50.88	36.84	−1.76	.07
Game performance in penetrating the defense:						
Pass decision-making	84.20	27.01	82.36	15.56	−0.61	.53
Dribbling decision-making	74.32	31.72	62.44	41.27	−1.29	.19
Get-free movements decision-making	91.30	12.3	79.94	19.57	−1.52	.12
Pass execution	62.14	30.65	60.62	23.42	−0.62	.53
Dribbling execution	86.94	24.13	77.33	36.73	−0.51	.61
Get-free movements execution	79.39	23.08	74.63	17.13	−1.11	.26
